# Quercetin Inhibits Angiogenesis Mediated Human Prostate Tumor Growth by Targeting VEGFR- 2 Regulated AKT/mTOR/P70S6K Signaling Pathways

**DOI:** 10.1371/journal.pone.0047516

**Published:** 2012-10-18

**Authors:** Poyil Pratheeshkumar, Amit Budhraja, Young-Ok Son, Xin Wang, Zhuo Zhang, Songze Ding, Lei Wang, Andrew Hitron, Jeong-Chae Lee, Mei Xu, Gang Chen, Jia Luo, Xianglin Shi

**Affiliations:** 1 Graduate Center for Toxicology, College of Medicine, University of Kentucky, Lexington, Kentucky, United States of America; 2 Department of Internal Medicine, College of Medicine, University of Kentucky, Lexington, Kentucky, United States of America; Henry Ford Health System, United States of America

## Abstract

Angiogenesis is a crucial step in the growth and metastasis of cancers, since it enables the growing tumor to receive oxygen and nutrients. Cancer prevention using natural products has become an integral part of cancer control. We studied the antiangiogenic activity of quercetin using *ex vivo*, *in vivo* and *in vitro* models. Rat aortic ring assay showed that quercetin at non-toxic concentrations significantly inhibited microvessel sprouting and exhibited a significant inhibition in the proliferation, migration, invasion and tube formation of endothelial cells, which are key events in the process of angiogenesis. Most importantly, quercetin treatment inhibited *ex vivo* angiogenesis as revealed by chicken egg chorioallantoic membrane assay (CAM) and matrigel plug assay. Western blot analysis showed that quercetin suppressed VEGF induced phosphorylation of VEGF receptor 2 and their downstream protein kinases AKT, mTOR, and ribosomal protein S6 kinase in HUVECs. Quercetin (20 mg/kg/d) significantly reduced the volume and the weight of solid tumors in prostate xenograft mouse model, indicating that quercetin inhibited tumorigenesis by targeting angiogenesis. Furthermore, quercetin reduced the cell viability and induced apoptosis in prostate cancer cells, which were correlated with the downregulation of AKT, mTOR and P70S6K expressions. Collectively the findings in the present study suggest that quercetin inhibits tumor growth and angiogenesis by targeting VEGF-R2 regulated AKT/mTOR/P70S6K signaling pathway, and could be used as a potential drug candidate for cancer therapy.

## Introduction

Angiogenesis, the formation of new blood vessels from preexisting blood vessels, is a crucial step in the growth, progression, and metastasis of tumors [1], [2], which enables the growing tumor to receive oxygen and nutrients [3]. The angiogenic process involves the activation, proliferation, and migration of endothelial cells toward angiogenic stimuli produced by the tumor [4]. Inhibition of angiogenesis is currently perceived as one of the promising strategies in the treatment of cancer.

Angiogenesis involves a sequence of coordinated events initiated by the expression of angiogenic factors with their subsequent binding to its cognate receptors on endothelial cells. Vascular endothelial growth factor (VEGF), the most important angiogenic signal protein, that stimulates tumor neoangiogenesis by increasing mitogenic and survival properties of vascular endothelial cells [5], [6]. The specific action of the VEGF on the endothelial cells is mainly mediated by two types of receptor tyrosine kinases (RTKs), VEGFR-1 and VEGFR-2. Of the two receptors, VEGFR-2 plays a more important role in mediating the mitogenesis and permeability of endothelial cells. Activation of VEGFR-2 contributes to phosphorylation of multiple downstream signals including ERK, JNK, PI3K, AKT, P70S6K and p38MAPK that subsequently promote proliferation, migration, and tube formation of endothelial cells [7].

The mammalian target of rapamycin (mTOR) is a protein kinase of the PI3K/Akt signalling pathway with a central role in the control of cell proliferation, survival, mobility and angiogenesis. Dysregulation of mTOR pathway has been found in many human tumours; therefore, the mTOR pathway is considered an important target for the development of new anticancer drugs [8]. One of the functions of Akt is phosphorylation and activation of mTOR. Subsequently, activated mTOR regulates p70S6K phosphorylation and activation [9]. The Akt-mTOR-p70S6K signaling pathway has been considered not only a central regulatory pathway of the protein translation involved in regulating cell proliferation, growth, differentiation and survival, but also a crucial step leading to angiogenesis in the neoplastic and non-neoplastic process [10].

**Figure 1:**
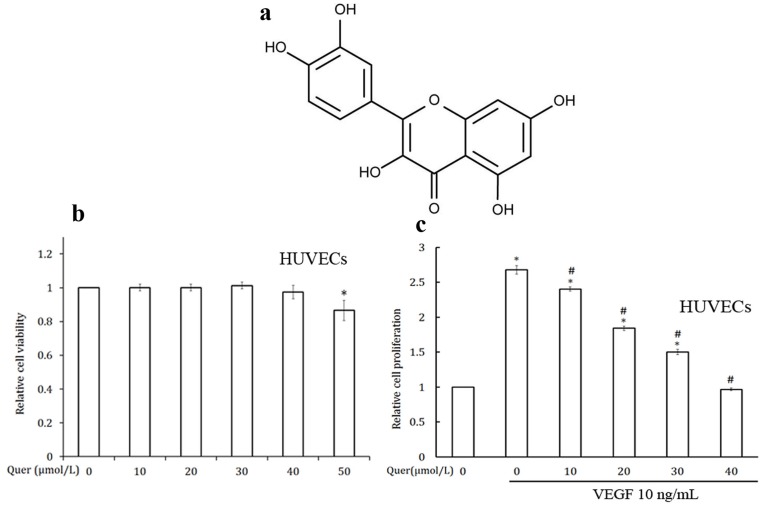
Quercetin inhibits the VEGF induced cell proliferation in HUVECs. (a) Chemical structure of quercetin. (b) Effect of quercetin on HUVECs viability in culture. HUVECs (5000 cells/well) were plated in a 96 well titer plate with different concentrations of quercetin and incubated for 48 h. Relative cell viability was determined by MTT assay. Values are means ± SD (mean of triplicate). *p<0.05 denotes a statistically significant difference from untreated controls. (c) Quercetin inhibits the VEGF induced proliferation of endothelial cells. HUVECs (5000 cells/well) in 96-well flat bottomed titer plate with different concentrations of quercetin and VEGF and incubated for 24 h. Relative cell proliferation was determined by MTT assay. Values are means ± SD (mean of triplicate). *p<0.05 denotes a statistically significant difference from untreated controls; #p<0.05 denotes a statistically significant difference from VEGF control.

Natural products are a tremendous source of active therapeutic agents, including anticancer agents. Cancer prevention using natural products has become an integral part of cancer control. Phytochemicals are potential novel leads for developing antiangiogenic drugs [11], [12]. Flavonoids are polyphenolic substances, widely distributed in almost every food plant, that possess antiviral, antimicrobial, anti-inflammatory, anti-allergic, anti-thrombotic, antimutagenic, antineoplasic, and cytoprotective effects on different cell types, both in animal and human models [13]. Epidemiologic studies have suggested that high consumption of flavonoids may be associated with decreased risk of several types of cancer [14]. Quercetin (Quer) (Fig. 1a) is found in a variety of plant-based foods such as red onions, apples, tea (*Camelia sinensis*), broccoli, capers, lovage, parsley, red grapes and a number of berries [15]. The potential chemopreventive effects of quercetin have been attributed to various mechanisms including its anti-oxidative activity as well as its capacity to inhibit enzymes that activate carcinogens, to modify signal transduction pathways, and to interact with and regulate cell receptors and other proteins [16]. In the present study, we analyzed the effect of quercetin on the inhibition of tumor specific angiogenesis *in vivo* as well as *in vitro* models. We found that quercetin can inhibit VEGF induced chemotactic migration, invasion, proliferation, and tube formation of HUVECs by suppressing VEGFR-2-regulated AKT/mTOR/P70S6K activation. Quercetin also blocks micro-vessel out growth in rat aortic ring and vascular density in CAM. Moreover, quercetin inhibits cancer growth and angiogenesis in human prostate xenograft mouse model.

## Materials and Methods

### Ethics Statement

Animals were handled in strict accordance with good animal practice as defined by Institutional Animal Care and Use Committee (IACUC), University of Kentucky (Approval ID: 2011-0851). The study was conducted adhering to the institutions guidelines for animal husbandry.

**Figure 2:**
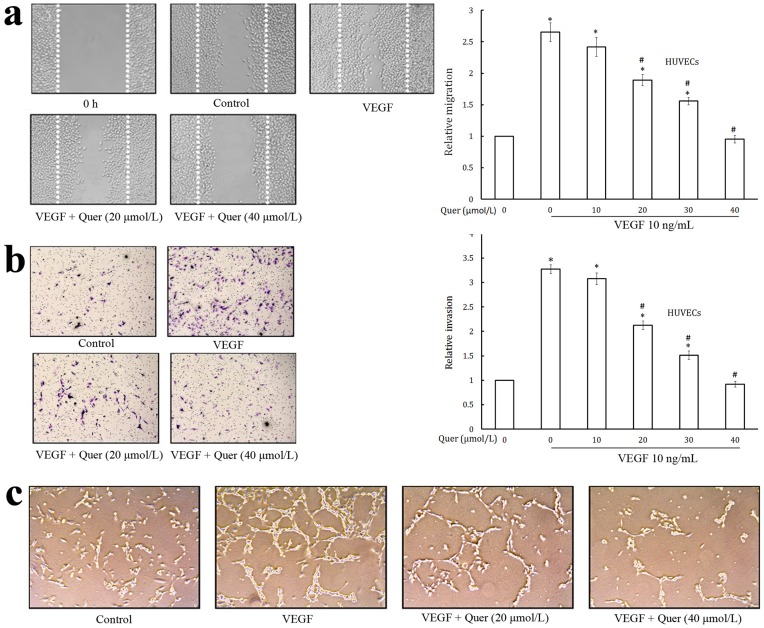
Quercetin inhibits VEGF-induced migration, invasion, and tube formation of endothelial cells. (a) Quercetin inhibited HUVECs migration. HUVECs were grown into wells of collagen coated 24 well plate dishes to 100% confluence. Cells were starved to inactivate cell proliferation and then wounded by pipette tips. EGM-2 containing 0.5% FBS was added with or without 10 ng/mL VEGF and different dilutions of quercetin. Migrated cells were quantified by manual counting. (b) Quercetin inhibited HUVECs invasion. HUVECs (10^5^ cells/Transwell) along with the indicated concentrations of quercetin were seeded into the upper compartment of invasion chambers. The bottom chambers were filled with EGM-2 supplemented with VEGF. After 24 h incubation, migrated cells were fixed, stained and quantified. (c) Quercetin inhibited the tube formation of HUVECs. HUVECs in medium EGM-2 were seeded into the matrigel layer in 24–well plates with VEGF. Various dilutions of quercetin were added into the wells and incubated for 24 h, cells were fixed, and tubular structures were photographed. Values are means ± SD (mean of triplicate). *p<0.05 denotes a statistically significant difference from untreated controls; #p<0.05 denotes a statistically significant difference from VEGF control.

### Chemicals and Reagents

Quercetin (>99% pure) was purchased from Sigma (St. Louis, MO, USA), dissolved in DMSO, aliquoted, and stored at −20°C. Bacteria-derived recombinant human VEGF (121 a.a.) was purchased from ProSpec-Tany TechnoGene Ltd. (Ness Ziona, Israel). Growth factor-reduced Matrigel was purchased from BD Biosciences (Bedford, MA). The antibodies anti-AKT, anti-mTOR, anti-p70S6K1, antipoly (ADP-ribose) polymerase (PARP), phospho-specific anti-AKT (Ser473), anti-mTOR (Ser2448), anti-p70S6K1 (Thr421/Ser424), and anti-VEGFR2 (Tyr1175) were purchased from Cell Signaling Technology (Beverly, MA). The antibody against β-actin was obtained from Santa Cruz Biotechnology (Santa Cruz, CA). Highly specific quantitative sandwich ELISA kit for human VEGF was purchased from RayBiotech (GA, USA).

**Figure 3:**
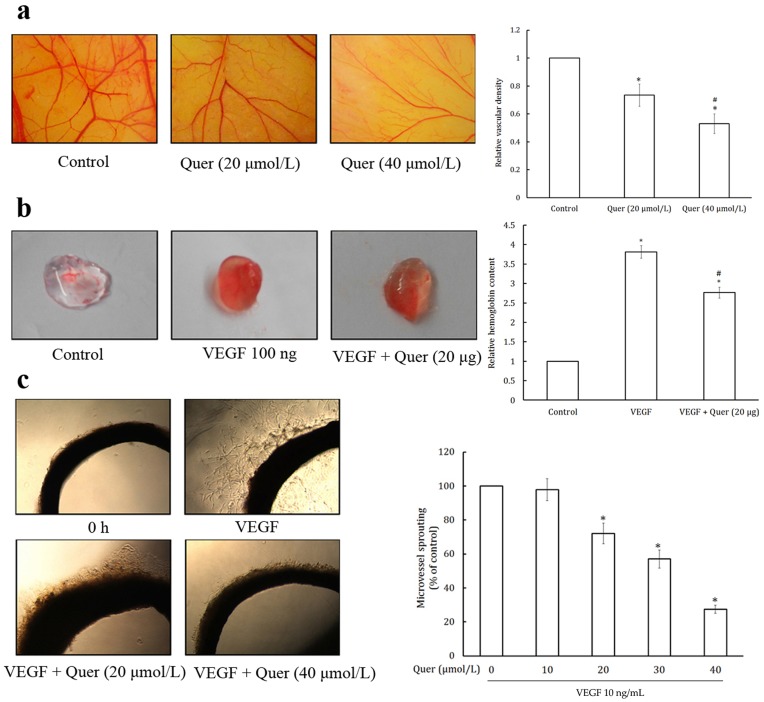
Quercetin inhibits *ex vivo* angiogenesis by CAM and matrigel plug assay and *in vitro* angiogenesis by rat aortic ring assay. (a) Quercetin inhibits *ex vivo* angiogenesis in CAM assay. Fertile leghorn chicken eggs were candled on embryonic day 8; a small opening was made at the top of the live eggs. Quercetin for treatment was mixed with 0.5% methyl cellulose in water and gently placed on the CAM. The eggs were incubated for 48 h and photographed. Blood vessels density was quantified by Image J software and represented as a bar diagram. Values are means ± SD (mean of triplicate). *p<0.05 denotes a statistically significant difference from untreated controls; #p<0.05 denotes a statistically significant difference from 20 and 40µmol/L quercetin. (b) Quercetin inhibits *ex vivo* angiogenesis in matrigel plug assay. Matrigel plug containg VEGF and quercetin were implanted into the CAM at day 9 of fertilized chicken eggs. After 96 h of incubation, the matrigel plugs were taken out and dispersed in PBS and incubated at 4°C overnight. Hemoglobin levels were determined using Drabkin’s reagent according to manufacturer instructions. Values are means ± SD (mean of triplicate). *p<0.05 denotes a statistically significant difference from untreated controls; #p<0.05 denotes a statistically significant difference from VEGF control. (c) Quercetin inhibits microvessel outgrowth from the rat aortic ring. Dorsal aorta from a freshly sacrificed Sprague–Dawley rat was taken out in a sterile manner and rinsed in ice cold PBS. It was then cut into ∼1 mm long pieces using surgical blade. Each ring was placed in a collagen pre-coated 96-well plate. VEGF, with or without different dilutions of quercetin, was added to the wells. On day 6, the rings were analyzed by phase-contrast microscopy and microvessel outgrowths were quantified and photographed. Values are means ± SD (mean of triplicate). *p<0.05 denotes a statistically significant difference from untreated controls.

### Cell Lines and Cell Culture

Human umbilical vein endothelial cells (HUVECs) were provided by Dr. Jia Luo (Department of Internal Medicine, University of Kentucky College of Medicine, Lexington, KY) and grown in Clonetics Endothelial Cell Growth Medium-2 (EGM-2; Lonza, Walkersville, MD) [17]. Human prostate cancer (PC-3) cells were purchased from American Type Culture Collection and cultured in RPMI 1640 medium supplemented with 10% fetal bovine serum (FBS). HUVECs and PC-3 cells were cultured at 37°C under a humidified 95%: 5% (v/v) mixture of air and CO_2_.

**Figure 4:**
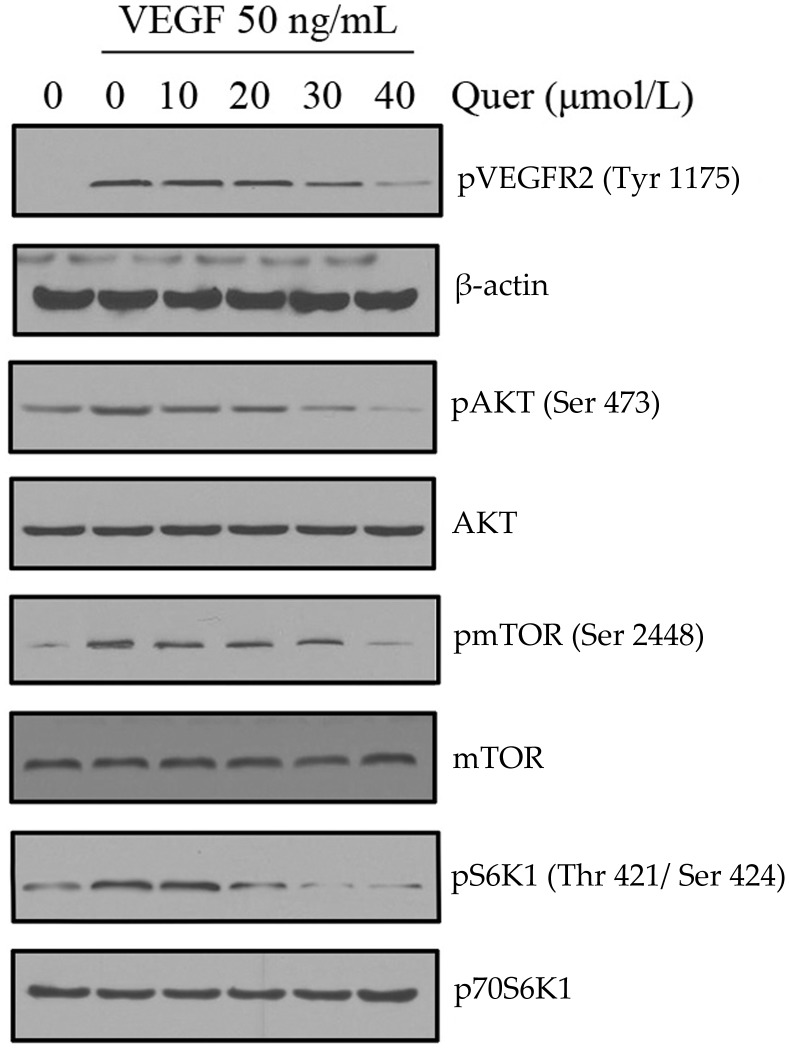
Quercetin inhibits the activation of VEGFR2-mediated signaling pathways in HUVECs. Quercetin suppressed the activation of VEGFR2 and their down stream AKT/mTOR/p70S6K pathway triggered by VEGF in HUVECs. Proteins from different treatments was tested by western blotting and probed with specific antibodies. Experiments were repeated for three times.

### MTT Assay

The MTT assay was employed to determine the number of viable cells in culture. Briefly, HUVECs or PC-3 cells were seeded (5000 cells/well) in 96-well flat bottomed titer plate and incubated for 24 h at 37°C in 5% CO_2_ atmosphere. Different dilutions of quercetin were added and incubated further for 24 h. Before 4 h completion of incubation, 10 µl MTT (5 mg/ml) was added [18]. The cultures were solubilized and spectrophotometric absorbance was measured at 595 nm using a microtiter plate reader. The number of viable cells was presented relative to untreated controls.

**Figure 5:**
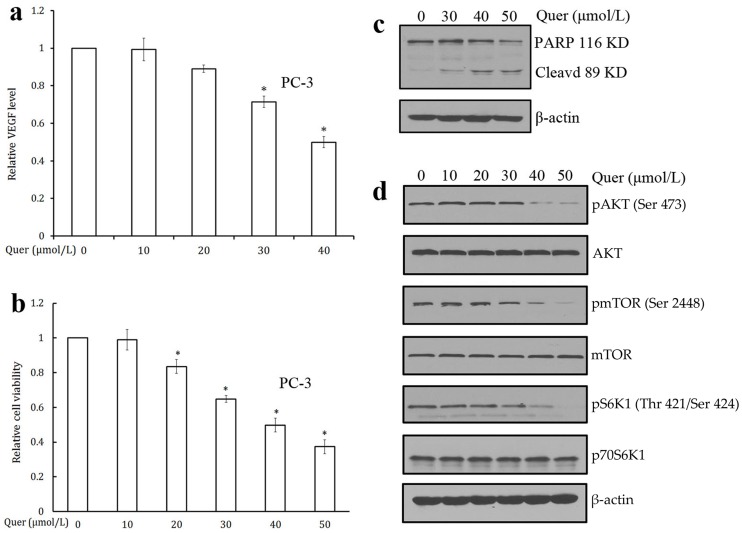
Quercetin induces cell apoptosis and inhibits the activation of AKT/mTOR/p70S6K pathway in prostate cancer cells. (a) Quercetin inhibited VEGF secretion in prostate PC-3 cancer cells. VEGF level was estimated by ELISA method. Values are means ± SD (mean of triplicate). *p<0.05 denotes a statistically significant difference from untreated controls. (b) Quercetin inhibited cell viability of prostate PC-3 cancer cells. Cell viability was quantified by MTT assay. Values are means ± SD (mean of triplicate). *p<0.05 denotes a statistically significant difference from untreated controls. (c) Quercetin induced PC-3 cancer cell apoptosis by the cleaved-PARP analysis. PC-3 cells were treated with quercetin for 48 h, and whole cell proteins were analysed by Western blotting with antipoly (ADP-ribose) polymerase (PARP). (d) Quercetin inhibited the activation of AKT/mTOR/p70S6K pathway in PC-3 cells. Proteins from different treatments was tested by western blotting and probed with specific antibodies. Experiments were repeated for three times.

### Cell Proliferation Assay

HUVECs were seeded (5000 cells/well) in 96-well flat bottomed titer plate and incubated for 24 h at 37°C in 5% CO_2_ atmosphere. EGM-2 (0.5% FBS) containing 10 ng/mL VEGF with or without different dilutions of quercetin was added and incubated for 24 h. Relative cell proliferation was determined by MTT assay.

**Figure 6:**
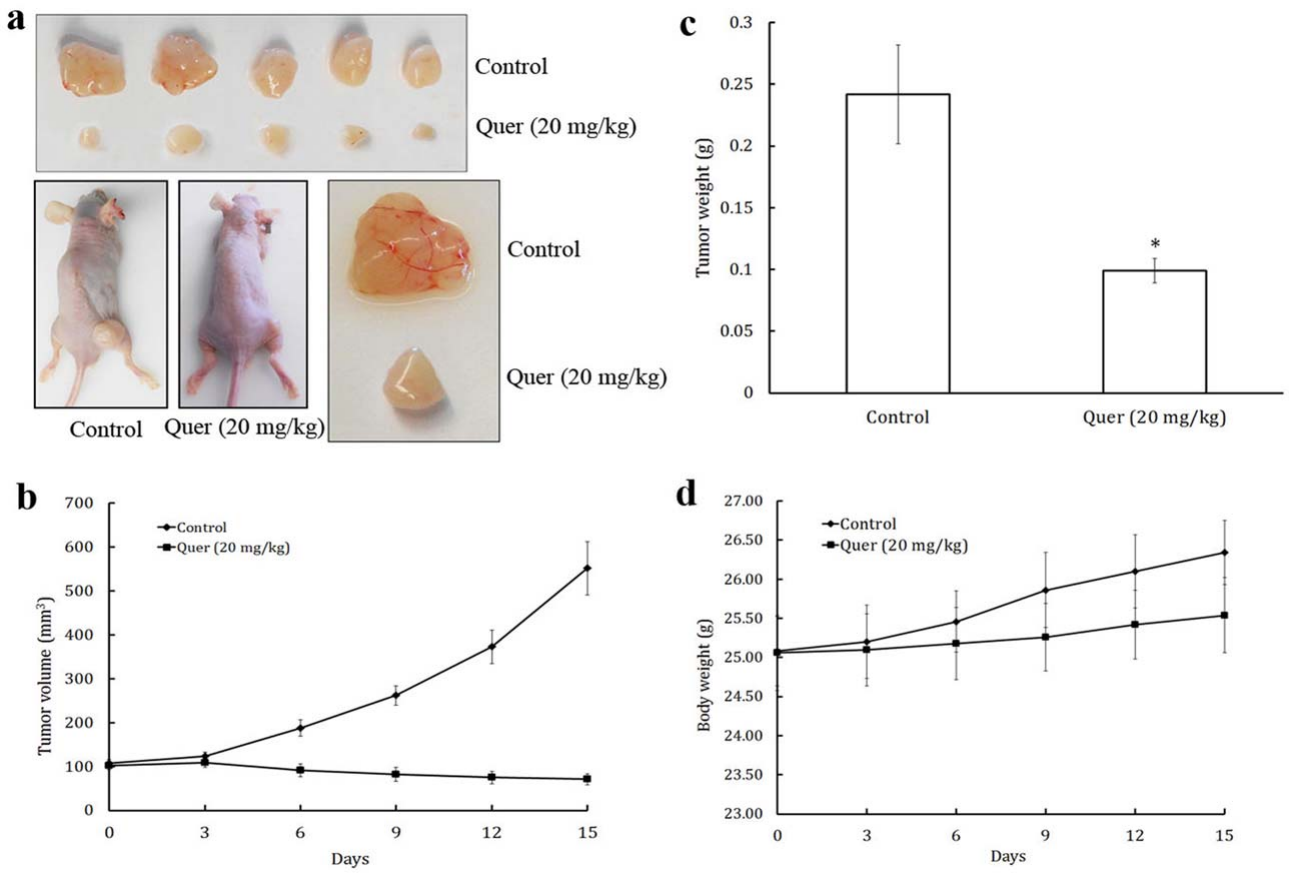
Quercetin inhibits tumor growth in a xenograft mouse model. PC-3 cells were injected into 6-week old BALB/cA nude mice (5×10^6^ cells per mouse). After tumors grew to about 100 mm^3^, mice were treated intraperitoneally with or without quercetin (20 mg/kg/d). (a) Solid tumors in the quercetin treated mice were significantly smaller than those in the control mice. Quercetin significantly reduced (b) tumor volume, and (c) tumor weight, (d) but had no effect on the body weight of mice. Values are means ± SD (mean of triplicate). *p<0.05 denotes a statistically significant difference from untreated controls.

### Wound-healing Migration Assay

HUVECs were grown into wells of collagen coated 24 well plate dishes to 100% confluence. Cells were starved to inactivate cell proliferation and then wounded by pipette tips. EGM-2 containing 0.5% FBS was added with or without 10 ng/mL VEGF and different dilutions of quercetin. Images of the cells were taken after 24 h of incubation. Migrated cells were quantified manually, and presented relative to untreated controls. Three independent experiments were performed.

**Figure 7:**
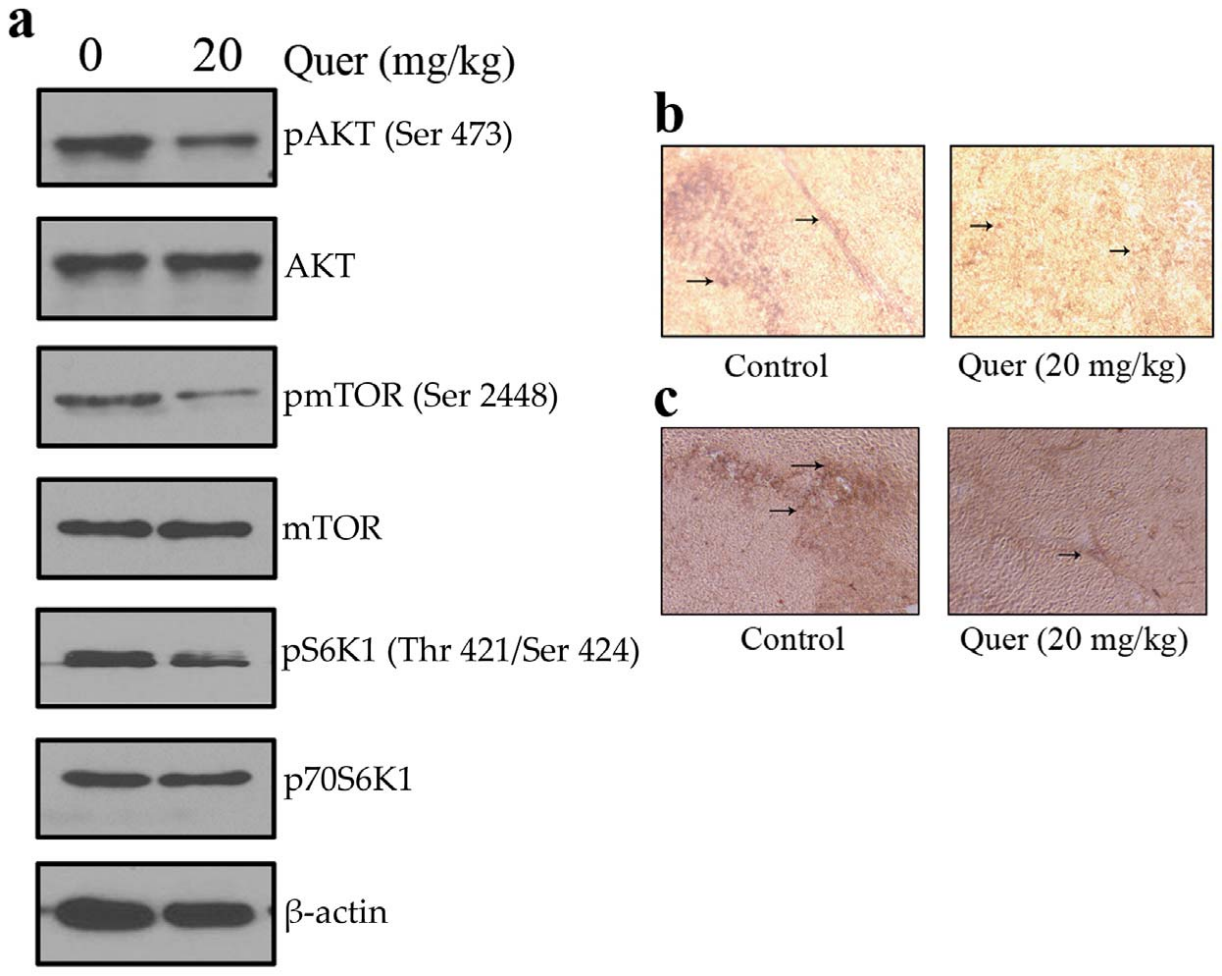
Quercetin inhibits tumor angiogenesis *in vivo* by suppressing AKT/mTOR/p70S6K pathway. (a) Quercetin inhibited the activation of AKT/mTOR/p70S6K pathway *in vivo*. Proteins from tumor tissue was tested by western blotting and probed with specific antibodies. Experiments were repeated for three times. Quercetin inhibited tumor angiogenesis as evident from (b) CD31 and (c) CD34 immunohistochemistry. Tumor sections (5 µm) were incubated with a rabbit anti-CD31 and mouse anti-CD34 antibodies and were subsequently incubated with biotinylated anti-rabbit/anti-mouse secondary antibody, followed by staining with Vectastain ABC Kit.

### Transwell Invasion Assay

Transwell invasion assay was performed as described previously [19]. Briefly, HUVECs (10^5^ cells/Transwell) along with the indicated concentrations of quercetin were seeded into the upper compartment of invasion chambers. The bottom chambers were filled with 500 µL EGM-2 supplemented with 10 ng/mL VEGF. After 24 h incubation, migrated cells were fixed with 3.7% paraformaldehyde and stained with 0.5% crystal violet in 2% ethanol. Membranes were washed and the dye was eluted with 10% acetic acid. Absorbance was measured at 595 nm using a microtiter plate reader (Beckman coulter). The number of invaded cells was presented relative to untreated controls.

### Capillary-like Tube Formation Assay

HUVECs in medium EGM-2 were seeded into the matrigel layer in 24–well plates at a density of 6×10^4^ cells/well along with 10 ng/ml VEGF. Various dilutions of quercetin were added into the wells and incubated for 24 h at 37°C in 5% CO_2_ atmosphere. Tube formation was examined and photographed using an inverted microscope (20X) [20].

### ELISA Assays for Secretion of VEGF

PC-3 cells (2×10^5^) were plated in 24-well plates and allowed to attach by overnight incubation at 37°C. Cells were treated with desired concentrations of quercetin for 24 h. Subsequently, the culture medium was collected and used to determine secretion of VEGF using commercially available kits according to the manufacturers’ recommendations.

### Rat Aortic Ring Assay

The rat aortic ring assay was used as the *in vitro* angiogenesis study model [21]. Dorsal aorta from a freshly sacrificed Sprague–Dawley rat was taken out in a sterile manner and rinsed in ice cold PBS. It was then cut into ∼1 mm long pieces using surgical blade. Each ring was placed in a collagen pre-coated 96-well plate. VEGF, with or without different dilutions of quercetin, was added to the wells. On day 6, the rings were analyzed by phase-contrast microscopy and microvessel outgrowths were quantified and photographed [22]. The assay was scored from 0 (least positive) to 5 (most positive) in a double-blind manner. Each data point was assayed 6 times [23].

### CAM Assay in Fertilized Chicken Eggs

The effect of quercetin on *ex vivo* angiogenesis was determined by CAM assay. Briefly, fertile leghorn chicken eggs (Poultry Breeding farm, University of Kentucky) were candled on embryonic day 8; a small opening was made at the top of the live eggs. Quercetin for treatment was mixed with 0.5% methyl cellulose in water and gently placed on the CAM. The eggs were incubated for 48 h and photographed. Blood vessels density was quantified by Image J software and represented as a bar diagram.

### Matrigel Plug Assay

Fertilized chicken eggs (Poultry Breeding farm, University of Kentucky) were incubated at 37°C for 9 days, and angiogenesis assay was performed as previously described [24]. In brief, VEGF (100 ng) and quercetin (20 µg) were mixed with matrigel and implanted into the CAM at day 9. After 96 h of incubation, the tumor plugs were taken out and dispersed in PBS and incubated at 4°C overnight. Hemoglobin levels were determined using Drabkin’s reagent (Sigma-Aldrich, St. Louis, MO) according to manufacturer instructions.

### Western Blot Analysis

To determine the effects of quercetin on the VEGFR2–dependent mTOR signaling pathway, HUVECs were first starved in serum-fee EGM-2 for 6 h, and then pretreated with or without quercetin for 1 h, followed by the stimulation with 50 ng/mL of VEGF for 10 min (for VEGFR2 activation) or 30 min (for mTOR pathway kinase activation). To examine mTOR pathway in prostate tumor cells, normal cultured PC-3 cells were directly treated with indicated dilutions of quercetin for 6 h. The total cellular samples were washed twice with ice-cold PBS and lysed in 1X NuPAGE LDS sample buffer supplemented with 50 mM dithiothreitol. The protein concentration was determined using Coomassie Protein Assay Reagent (Pierce, Rockford, IL). About 40 µg cellular proteins were separated using 6%–12% SDS-polycrylamide gel and transferred to nitrocellulose membrane. Membranes were blocked with 5% fat-free dry milk in 1X Tris-buffered saline (TBS) and incubated with antibodies. Protein bands were detected by incubating with horseradish peroxidase-conjugated antibodies (Kirkegaard and Perry Laboratories, Gaithersburg, MD) and visualized with enhanced chemiluminescence reagent (Perkin Elmer, Boston, MA).

For tissue sections, radioimmunoprecipitation assay (RIPA) buffer was added to the sections and homogenized with electric homogenizer. After incubation for 20 minutes on ice, samples were centrifuged for 30 minutes at 12,000 rpm at 4°C and supernatant was collected as total cell lysate. SDS-PAGE was carried out as described previously [25].

### Xenograft Human Prostate Tumor Mouse Model

Six week old male BALB/cA nude mice were purchased from charles River Laboratories (Wilmington, MA). Animals were housed in a specific pathogen-free room within the animal facilities at the University of Kentucky, Lexington, KY. All animals were allowed to acclimatize to their new environment for one week prior to use and were handled according to the Institutional Animal Care and Use, University of Kentucky. Mice were randomly divided into 2 groups (5 animals/group). PC-3 cells (5×10^6^ cells per mouse) were resuspended in serum-free RPMI-1640 medium with matrigel basement membrane matrix (BD Biosciences) at a 1∶1 ratio (total volume: 100 µL) and then were subcutaneously injected into the flanks of nude mice. After tumors grew to about 100 mm^3^, mice were treated intraperitoneally with or without quercetin (20 mg/kg/d). The body weight of each mouse was recorded and tumor volume was determined by Vernier caliper every day, following the formula of *A×B^2^×0.52*, where A is the longest diameter of tumor and B is the shortest diameter. After 16 d, the mice were killed by cervical dislocation and solid tumors were removed.

### Histology and Immunohistochemistry

Tumor tissues were fixed in 10% neutral-buffered formalin for 24 hours, processed, and embedded in paraffin blocks. The sections (5 µm) were blocked with 10% goat serum and incubated with a rabbit anti-CD31 (1∶100; Novus Biologicals Inc, Littleton, CO) and mouse ant-CD34 (1∶100; BD Pharmingen Inc, San Diego, CA) antibodies for 24 h. The slides were subsequently incubated for 30 min with biotinylated anti-rabbit/anti-mouse secondary antibody (Vector laboratories, Burlingame, CA) and followed by incubation of Vectastain ABC Kit (Vector Laboratories). Diaminobenzidine (Sigma) was used as the chromagen and methyl green (Sigma) as the counterstain.

### Statistics

The values were presented as means ± SD. Two-way analysis of variance (ANOVA) and Student’s t test were used for statistical analysis. p<0.05 was considered significantly different.

## Results

### Effect of Quercetin Towards HUVECs Viability in Culture

Cell viability was determined by MTT assay. Effect of quercetin on HUVECs viability in culture is shown in Figure 1b. At concentrations of 10–40 µmol/L quercetion was found to be non-toxic to HUVECs and these concentrations were used for further *in vitro* experiments.

### Quercetin Inhibits HUVECs Proliferation, Chemotactic Migration, Invasion, and Tube Formation

VEGF plays an important role during neo-angiogenesis through its mitogenic effect on endothelial cells [26]. HUVECs showed very high rate of proliferation when stimulated with VEGF. Quercetin treatment at concentrations of 10-40 µmol/L significantly inhibited VEGF induced proliferation of HUVECs (Fig. 1c).

Effect of quercetin on the chemotactic motility of HUVECs is shown in Figure 2a. HUVECs migrated into the clear area when stimulated with chemoattractant, VEGF. Quercetin significantly inhibited the VEGF induced migration of endothelial cells in a dose dependent manner and maximum inhibition of endothelial cell migration was observed at 40 µmol/L and was almost similar to that of zero hour incubation. This concentration is non-toxic as is evident from MTT assay (Fig. 1b) and hence the inhibitory effect could not be attributed to cytotoxic activity. HUVECs showed a high invasive property through the collagen matrix when stimulated with VEGF (Fig. 2b). Treatment of quercetin produced a significant inhibition in the invasion of the collagen matrix by HUVECs in a dose dependent manner. The tubular formation of endothelial cells is also a key step of angiogenesis [27]. Treatment of HUVECs with quercetin significantly inhibited tube formation (Fig. 2c). Incubation of HUVECs on matrigel with VEGF resulted in the formation of elongated and tube like structures. Quercetin effectively reduced the width and length of endothelial tubes at 20 and 40 µmol/L.

### Quercetin Inhibits *ex vivo* Angiogenesis in CAM Assay

CAM assay was used to determine the antiangiogenic effect of quercetin *ex vivo*. CAM revealed highly vascularized structure in the control group (Fig. 3a). Exposure to quercetin (20 and 40 µmol/egg) drastically reduced the vascular density. These results confirmed the anti-angiogenic potential of quercetin through an *ex vivo* assay.

### Quercetin Inhibits *ex vivo* Angiogenesis in Matrigel Plug Assay

To confirm the anti-angiogenesis effects of quercetin *ex vivo*, matrigel plug assay was performed. As shown in Figure 3b, quercetin (20 µg) significantly inhibited VEGF-induced angiogenesis in the matrigel plug, indicating quercetin effectively inhibited angiogenesis *ex vivo*. Hemoglobin level was also significantly lower in the quercetin treated matrigel plug, further confirming their antiangiogeneic potential.

### Quercetin Inhibits Microvessel Outgrowth from the Rat Aortic Ring

To study the inhibitory effect of quercetin on *in vitro* angiogenesis, we performed aortic ring assay. VEGF can induce microvessel outgrowth in rat aorta ring. As shown in Figure 3c, quercetin at 20 and 40 µmol/L inhibited micro-vessel growth after 6 days incubation, indicating that quercetin inhibits angiogenesis *in vitro*.

### Quercetin Inhibits the Activation of VEGFR2-mediated Signaling Pathways in Endothelial Cells

VEGFR2 binds with VEGF that activates various downstream signaling molecules responsible for endothelial cell migration, proliferation, and survival. To understand the molecular mechanism of quercetin-mediated anti-angiogenic properties, we examined the signaling molecules and pathways using western blotting assays. VEGF treatment strongly increased the VEGFR2 phosphorylation at Ser1175 site, a reliable marker for its activity. In our study, we found that phosphorylation of VEGFR2 was suppressed by quercetin in a dose-dependent manner (Fig. 4). Quercetin significantly suppressed the activation of VEGFR2 downstream signaling molecules such as AKT, mTOR, and p70S6K, which indicated that quercetin inhibited angiogenesis through direct inhibition of VEGFR2 on the surface of endothelial cells. Extensive down regulation of phospho-AKT (Ser473), a well-known downstream target of VEGFR2, was observed at 40 µM quercetin, however total AKT levels remain unchanged (Fig. 4, upper panel western). Next, we examined the expression of phospho-mTOR (Ser2448) after quercetin exposure and the results in Figure 4 (lower panel western) revealed that phospho-mTOR levels were also decreased together with phospho-AKT. Total mTOR levels were unaltered. Furthermore, phospho-S6K (downstream target of mTOR) was decreased in a dose- dependent exposure in endothelial cells (Fig. 4, lower western). The concentrations of quercetin used for the above experiments were found to be non-toxic to endothelial cells (Fig. 1b), suggesting that the effect of quercetin on endothelial cells were not through decrease in cell viability. Collectively, the results described in this section indicated that quercetin inhibited VEGF mediated angiogenesis through VEGFR2 mediated pathway.

### Quercetin Inhibits VEGF Secretion in PC-3 Cells

VEGF plays an important role in angiogenesis by promoting endothelial cell proliferation, migration, and differentiation [28]. We determined the effect of quercetin on VEGF secretion and the results are shown in Figure 5a. The quercetin treatment caused a dose-dependent and statistically significant decrease in VEGF secretion into the medium.

### Quercetin Induces Cancer Cell Apoptosis and Inhibits AKT/mTOR/P70S6K Pathway in PC-3 Prostate Cancer Cells

Poly (ADP-ribose) polymerase (PARP) is a family of proteins involved in cell death [29], [30], and shown to be cleaved into 89- and 24-kD fragments that contain the active site and the DNA-binding domain of the enzyme, respectively, during drug induced apoptosis in a variety of cells [31]-[33]. Quercetin significantly decreased the PC-3 cell viability (Fig. 5b) and also induced tumor cell apoptosis by detecting full length PARP (116 kDa) and its large cleavage fragment (89 kDa) (Fig. 5c). This data suggest that quercetin also have direct cytotoxic effects on cancer cells besides its antiangiogenic effect on endothelial cells. To verify the inhibitory effect of quercetin on PC-3 cell viability, we further examined the effect of different concentrations of quercetin on the phosphorylation of AKT, mTOR, and P70S6K. As shown in Figure 5d, quercetin dramatically inhibited the phosphorylation of AKT, mTOR, and P70S6K, but the total protein levels remain unchanged, indicating that the AKT/mTOR pathway is also a possible target of quercetin in tumor cells.

### Quercetin Inhibits Tumor Angiogenesis and Tumor Growth *in vivo*

We used a xenograft prostate tumor model to investigate the effect of quercetin on tumor growth and angiogenesis. PC-3 prostate cancer cells were injected (5×10^6^ per mouse) into the 6-week-old male BALB/cA nude mice. After the tumors had developed (about 100 mm^3^), the mice were injected with or without 20 mg/Kg/day quercetin (ip) every day (Fig. 6a). We found that intraperitoneal administration of quercetin significantly suppressed tumor volume (Fig. 6b) and tumor weight (Fig. 6c) but had no effect on the body weight of mice (Fig. 6d). As shown in Figure 6b, tumors in control group increased from 108.31±7.35 to 551.66±61.32 mm^3^, whereas tumors in quercetin-treated group decreased from 101.77±8.57 to 71.16±2.65 mm^3^. The average weight of tumors from the control group was 0.242±0.04 gram whereas the average weight in quercetin treated group was only 0.099±0.01 gram, suggesting strong anti-tumor potential of quercetin in xenograft mouse prostate tumor model.

To further investigate whether quercetin inhibited tumor growth by suppressing tumor angiogenesis, we performed western blot and immunohistochemical analysis of solid tumors (Fig. 7). Tumors from quercetin treated animals showed a suppressed activation of AKT, mTOR and P70S6K proteins (Fig. 7a). We also observed a large number of CD31 (Fig. 7b) and CD34 (Fig. 7c) positive cells in untreated control group whereas a small number in quercetin treated group. All these observations indicate the antiangiogenic efficacy of quercetin *in vivo* that strongly support the above *ex vivo* and *in vitro* studies.

## Discussion

Tumors can grow up to ∼2 mm size without requirement of blood supply as diffusion is sufficient at this level to support the removal of wastes from and supply of nutrients to tumor cells. Therefore, angiogenesis process could be an important target to suppress tumor growth and metastasis. Angiogenesis is required at almost every step of tumor progression and metastasis, and tumor vasculature has been identified as strong prognostic marker for tumor grading [34]. So inhibition of angiogenesis induced by tumor and metastasis cells is a promising therapeutic strategy for cancer. Several antiangiogenic strategies have been developed to inhibit tumor growth by targeting different components of tumor angiogenesis. Many phytochemicals could have a tremendous potential as antiangiogenic agents to check the cancer development and metastasis [34].

Invasion, migration, proliferation and tube formation of endothelial cells are important steps in the angiogenic cascade. Treatment with quercetin dose dependently inhibited the collagen matrix invasion, chemotactic migration, proliferation and tube formation of HUVECs *in vitro.* Among many angiogenesis assays, the CAM assay is well established and widely used as a model to examine anti-angiogenesis [35]. In the present study, we demonstrated that quercetin significantly inhibited neovascularization *ex vivo* in CAM assay and matrigel plug assay. Quercetin also exhibited cytotoxicity and induced apoptosis towards prostate tumor cells (PC-3). We found that intraperitoneal administration of quercetin significantly suppressed volume and weight of tumors, but had no effect on the body weight of mice. Immunohistochemical data also showed that the expressions of endothelial cell markers, CD31 and CD34 were markedly less in tumor sections of quercetin treated animals.

Several angiogenic activators and inhibitors have been identified. Amongst the many proangiogenic mechanisms, the vascular endothelial growth factor (VEGF) signaling pathway has been implicated as the key regulator of tumor neovascularization [36]. VEGF is thus an attractive therapeutic target. VEGF has been demonstrated to have a major association with initiating the process of angiogenesis through regulating proliferation, migration, and differentiation of endothelial cells [37]. Here in our study, quercetin significantly inhibited the level of VEGF in PC-3 cells.

The mammalian target of rapamycin (mTOR) has been identified as a key player in tumor growth, metastasis and angiogenesis [38]. Dysregulation of mTOR pathway has been found in many human tumors; therefore, the mTOR pathway is considered an important target for the development of new anticancer drugs [8]. AKT is a serine/threonine kinase that plays a central role in a range of cellular functions including cell growth, proliferation, migration, protein synthesis, and angiogenesis [39], [40]. P70S6K kinase (p70S6K), a downstream of AKT, plays an important role in regulating tumor microenvironment and angiogenesis [41]. Recently, AKT/mTOR/p70S6K signaling has been identified as a novel, functional mediator in angiogenesis [23]. Treatment with quercetin showed a sharp decrease in the phosphorylation of mTOR and p70S6K, and its upstream kinase, AKT, suggesting that quercetin suppresses tumor angiogenesis by inhibiting VEGFR2 and blocking its multiple downstream signaling components. In conclusion, the present study shows that quercetin is a potent inhibitor of angiogenesis *in vitro, ex vivo* and *in vivo*. Quercetin treatment inhibited the activation of VEGF-R2 and thereby suppressed the AKT/mTOR/P70S6K mediated angiogenesis signaling pathways.
